# Eliminating Anti-Nutritional Plant Food Proteins: The Case of Seed Protease Inhibitors in Pea

**DOI:** 10.1371/journal.pone.0134634

**Published:** 2015-08-12

**Authors:** Alfonso Clemente, Maria C. Arques, Marion Dalmais, Christine Le Signor, Catherine Chinoy, Raquel Olias, Tracey Rayner, Peter G. Isaac, David M. Lawson, Abdelhafid Bendahmane, Claire Domoney

**Affiliations:** 1 Department of Physiology and Biochemistry of Animal Nutrition, Estación Experimental del Zaidín (CSIC), Profesor Albareda 1, 18008 Granada, Spain; 2 Unité de Recherche en Génomique Végétale (URGV), UMR INRA 1165—CNRS 8114—UEVE 2, Rue Gaston Crémieux—CP 5708—F-91000 Evry cedex, France; 3 UMR 1347 Agroécologie AgroSup/INRA/uB, Pôle Génétique & Ecophysiologie GEAPSI, 17 rue Sully BP 86510, 21065 Dijon cedex, France; 4 Department of Metabolic Biology, John Innes Centre, Norwich Research Park, Norwich NR4 7UH, United Kingdom; 5 IDna Genetics Ltd, Norwich Research Park, Norwich NR4 7UH, United Kingdom; 6 Department of Biological Chemistry, John Innes Centre, Norwich Research Park, Norwich NR4 7UH, United Kingdom; UMR INRA/INSA, BF2I, FRANCE

## Abstract

Several classes of seed proteins limit the utilisation of plant proteins in human and farm animal diets, while plant foods have much to offer to the sustainable intensification of food/feed production and to human health. Reduction or removal of these proteins could greatly enhance seed protein quality and various strategies have been used to try to achieve this with limited success. We investigated whether seed protease inhibitor mutations could be exploited to enhance seed quality, availing of induced mutant and natural *Pisum* germplasm collections to identify mutants, whilst acquiring an understanding of the impact of mutations on activity. A mutant (TILLING) resource developed in *Pisum sativum* L. (pea) and a large germplasm collection representing *Pisum* diversity were investigated as sources of mutations that reduce or abolish the activity of the major protease inhibitor (Bowman-Birk) class of seed protein. Of three missense mutations, predicted to affect activity of the mature trypsin / chymotrypsin inhibitor TI1 protein, a C77Y substitution in the mature mutant inhibitor abolished inhibitor activity, consistent with an absolute requirement for the disulphide bond C77-C92 for function in the native inhibitor. Two further classes of mutation (S85F, E109K) resulted in less dramatic changes to isoform or overall inhibitory activity. The alternative strategy to reduce anti-nutrients, by targeted screening of *Pisum* germplasm, successfully identified a single accession (*Pisum elatius*) as a double null mutant for the two closely linked genes encoding the TI1 and TI2 seed protease inhibitors. The *P*. *elatius* mutant has extremely low seed protease inhibitory activity and introgression of the mutation into cultivated germplasm has been achieved. The study provides new insights into structure-function relationships for protease inhibitors which impact on pea seed quality. The induced and natural germplasm variants identified provide immediate potential for either halving or abolishing the corresponding inhibitory activity, along with associated molecular markers for breeding programmes. The potential for making large changes to plant protein profiles for improved and sustainable food production through diversity is illustrated. The strategy employed here to reduce anti-nutritional proteins in seeds may be extended to allergens and other seed proteins with negative nutritional effects. Additionally, the novel variants described for pea will assist future studies of the biological role and health-related properties of so-called anti-nutrients.

## Introduction

Legume seeds are an excellent source of dietary protein but contain several protein classes which resist proteolysis to different degrees, retain biological activity during digestion due to their high level of stability and/or affinity for target enzymes or receptors, or are otherwise negatively associated with quality. *In vivo* studies have identified several of those protein classes resistant to digestion, including lectins, protease inhibitors and albumin proteins, which differ in type, abundance and relevance among legume species [1–5]. Here we have targeted the protease inhibitors, widespread among legume crops, with the aim of identifying mutations for fundamental studies of action mechanisms and with potential to enhance seed protein quality.

Protease inhibitors, specifically trypsin / chymotrypsin inhibitors (TI), in the seeds of legume crop species are regarded as a limitation to the exploitation of seeds, often leading to a requirement for heat-treatment of seed products during processing for feed uses [6]. The mode of activity of protease inhibitors involves the formation of a stoichiometric complex between the inhibitor and the target enzyme(s), mediated by an exposed binding loop inserted into the convex active site of the target protease in a substrate-like manner. The resulting non-covalent enzyme-inhibitor complex renders the protease(s) target inactive [7,8]. The development and exploitation of near-isogenic pea lines with distinct alleles at the *Tri* (trypsin inhibitor) locus controlling quantitative variation in protease inhibitory activity in pea seeds clearly demonstrated the correlation between allelic variants and amino acid availability of pea protein in poultry [9]. Pea seed TI are predominantly of the Bowman-Birk inhibitor (BBI) class, and qualitative and quantitative genetic variants have been described within a five-fold range of inhibitory activity [10]. Isoforms of the major pea seed-expressed BBI have been shown to be encoded by two genes, *TI1* and *TI2*, that are closely linked, and they inhibit both trypsin and chymotrypsin [11,12]. Minor pea BBI isoforms have predicted sites for trypsin inhibition only [12].

**Fig 1:**
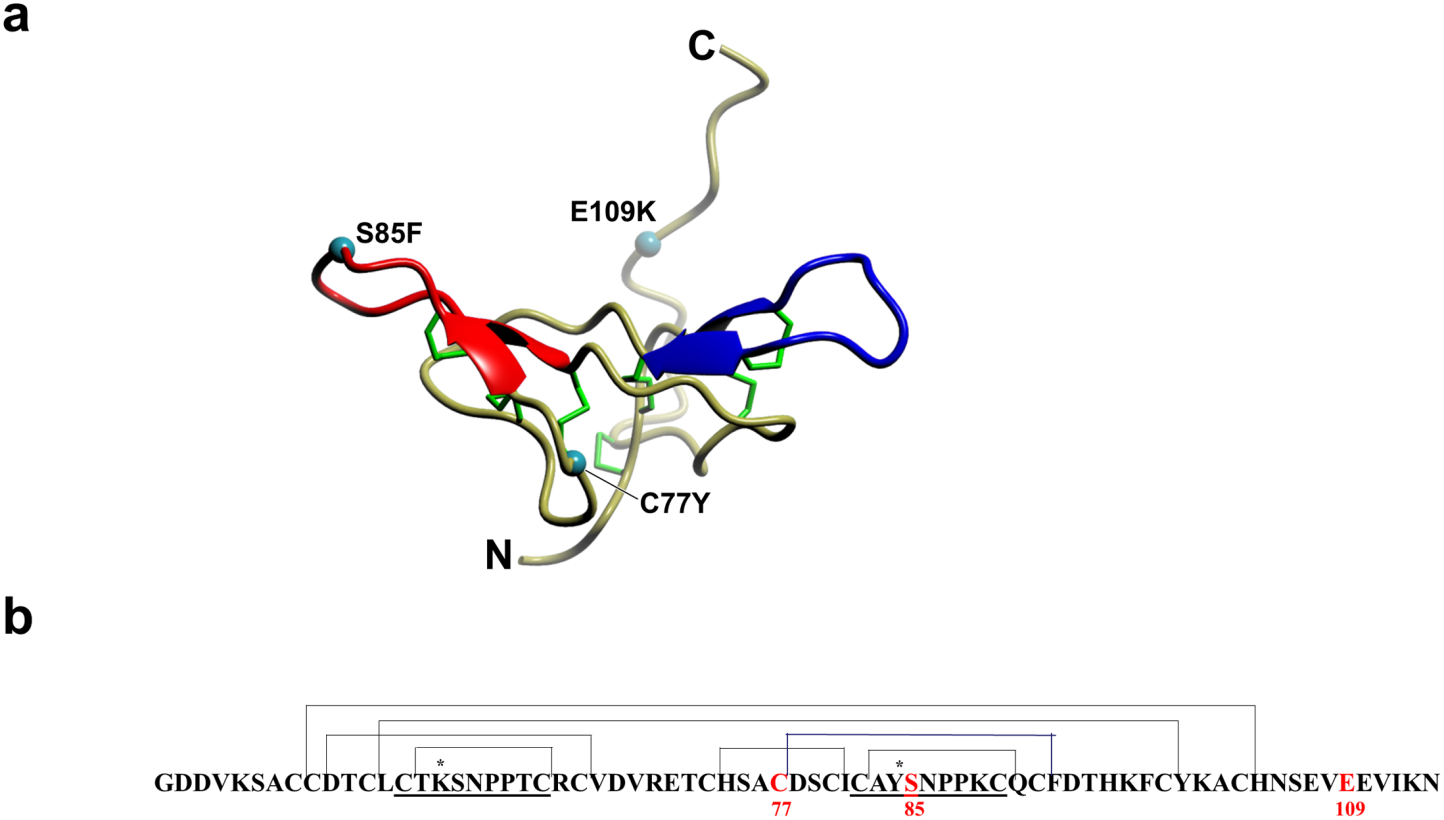
Impact of mutations on TI1 structure. (**a**) Homology model of TI1, a major Bowman-Birk inhibitor from pea. The trypsin (blue) and chymotrypsin (red) inhibitory domains are shown, with the identity and location of the mutations indicated by cyan spheres and the disulphide bonds in green. N and C refer to the amino- and carboxy-terminal ends, respectively. (**b**) Amino acid sequence deduced from the *TI1* gene from the pea cultivar Cameor. The sequences of the inhibitory domains are underlined and the positions of the seven disulphide bonds are indicated with connecting lines. The disulphide bond affected by the mutation C77Y is highlighted in blue. K and Y at position P1 (*) determine specificity for trypsin and chymotrypsin, respectively. Letters and numbers in red indicate the positions of the mutations shown in (a). Amino acid numbers are based on the protein coding region of the gene, which includes a 42 amino acid pre-pro-peptide; carboxy-terminal processing removes the last nine amino acids from a subset of TI proteins in vivo.

The BBI proteins show considerable variation between and within species, where seed and vegetative isoforms may be distinguished [11,12]. The expression of distinct genes, post-translational modification and differences in the oligomeric state of the inhibitors, are responsible for intra-specific variation and these may act in combination to affect inhibitory properties [13]. The BBI are synthesised as precursors of approximately 100 amino acid residues, giving rise to mature proteins with a molecular weight in the range 6000–9000. Mature BBI contain two protease binding loops, located at opposite sides of the molecule, stabilised by a characteristic highly conserved array of disulphide bridges involving 14 cysteine residues (see Fig 1, C50-C103, C51-C66, C54-C99, C56-C64, C73-C80, C77-C92 and C82-C90, amino acid numbering according to predicted pre-pro-protein). In combination, the disulphide bonds are likely responsible for the stability of BBI towards extreme conditions (high temperature, acid pH and attack of proteolytic enzymes) and for maintaining the structural and functional features of the binding sites [14,15].

In order to gain knowledge of the structure-function relationships within protease inhibitors and their variants, and to enhance seed quality, natural variants and mutations have been sought in a number of species with some success. Null mutants or variants for seed protease inhibitors (Kunitz, which is distinct from BBI, and some BBI) have been described in *Glycine max* (soybean) and *Glycine soja* (wild soja) [16–20]. An alternative approach to reducing seed inhibitor activity has generated transgenic lines of soybean expressing a mutant *BBI* transgene where both active sites have been interrupted with an inserted Gly residue. These lines showed a significant reduction in the amount of seed inhibitor overall (activity reduced by 20 to 50%), likely as a consequence of the earlier expression of the transgene and a limited available sulphur amino acid pool [20]. Soybean lines having combinations of these and additional mutations and around 15% of wild-type inhibitor activity are the subject of ongoing patent claims [21,22].

The availability of mutagenized resources in crop plants is accelerating the discovery of desired mutations affecting seed quality and facilitates fundamental studies of such mutations alongside establishing their pleiotropic effects on plant performance. Equally, high-throughput screening methods facilitate the exploitation of such resources and germplasm collections representing broad genetic diversity. Here we adopted two approaches to identify and study the effects of mutations that impact on the accumulation of the major seed inhibitors in *Pisum sativum* L. (pea). The first approach exploited a TILLING (Targeted Induced Local Lesions IN Genomes) mutagenized resource, which has yielded a number of alleles for fundamental studies and has provided insights into the structure-function relationships of the targeted protein. The second approach involved targeted screening of the wider *Pisum* germplasm [23] to identify novel genetic variants. Both approaches have been successful in identifying mutations which have been characterised for their impact on inhibitor activity, and in delivering novel germplasm that can be exploited for improved seed products. The study has revealed the huge potential for making the large changes that are often desired in plant protein profiles through exploiting diversity, both natural and induced.

## Results

The development of a TILLING platform for functional genomics in *Pisum sativum* L. has been described and its utility demonstrated [24–26]. Here screening for mutations in the *TI1* gene of pea, encoding one of two major seed protease inhibitors, identified a total of 13 nucleotide changes; of these seven were in non-coding regions (four upstream of the 5’ untranslated region and three downstream of the stop codon). Of the six changes affecting the coding sequence, two were silent and one missense mutation in the pre-pro-peptide region (T33I) was not investigated further. Null mutations were not identified; the probability of isolating a null mutation was reduced since *TI1* is an intron-less gene and gene variants capable of generating mis-spliced transcripts were not expected. The three missense mutations within the mature protein were predicted to impact on the function of the encoded inhibitor, affecting amino acid residues involved in: one of the intramolecular disulphide bonds (C77Y), the chymotrypsin inhibitory active site (S85F), and the carboxy-terminal region (E109K) that is removed from a subset of mature inhibitors *in vivo* [27] (Table 1, Fig 1). The C77Y mutation was predicted to impact on one of the disulphides involved in stabilising the chymotrypsin inhibitory loop (C77-C92, Fig 1); the S85F mutation was predicted to impact on the chymotrypsin inhibitory activity whereas the E109K mutation was hypothesised not to impact seriously on inhibitory activity (Fig 1) but could potentially influence dimerization of the inhibitor, in which the carboxy-terminal domain has a suggested role [28].

**Table 1:** Missense *TI1* gene mutants obtained by TILLING and used in this study.

*TI1* mutation	TI1 mutation	Family	Mutant	Wild type
G230A	C77Y	2808	2/1/3	1/1/4
			2/1/6	1/1/6
C254T	S85F	671	19/4	19/6
			19/11	19/7
			19/19	19/13
			19/24	
G325A	E109K	895	1/1/15	1/2/6
			1/2/4	1/1/14
			1/2/9	1/2/7

Changes in encoded proteins are shown, and the BC2F3 and BC2F4 lineage identifiers for mutant and corresponding wild-type mutant alleles. Mutation positions for genes and proteins are given relative to the initiator methionine codon or amino acid, respectively.

Mutant and wild-type segregants were selected from backcrosses (BC) and bulked BC2F3 and BC2F4 seeds of validated mutant and wild-type lines of the three families used for biochemical assays. Analysis of total seed protein and albumin profiles by protein gel electrophoresis and measurement of the amounts of these protein fractions did not reveal any significant difference between the mutant and wild-type lines within any one family (S1a–S1d Fig).

### Inhibitory activities are reduced differentially by the mutations in TI1

**Fig 2:**
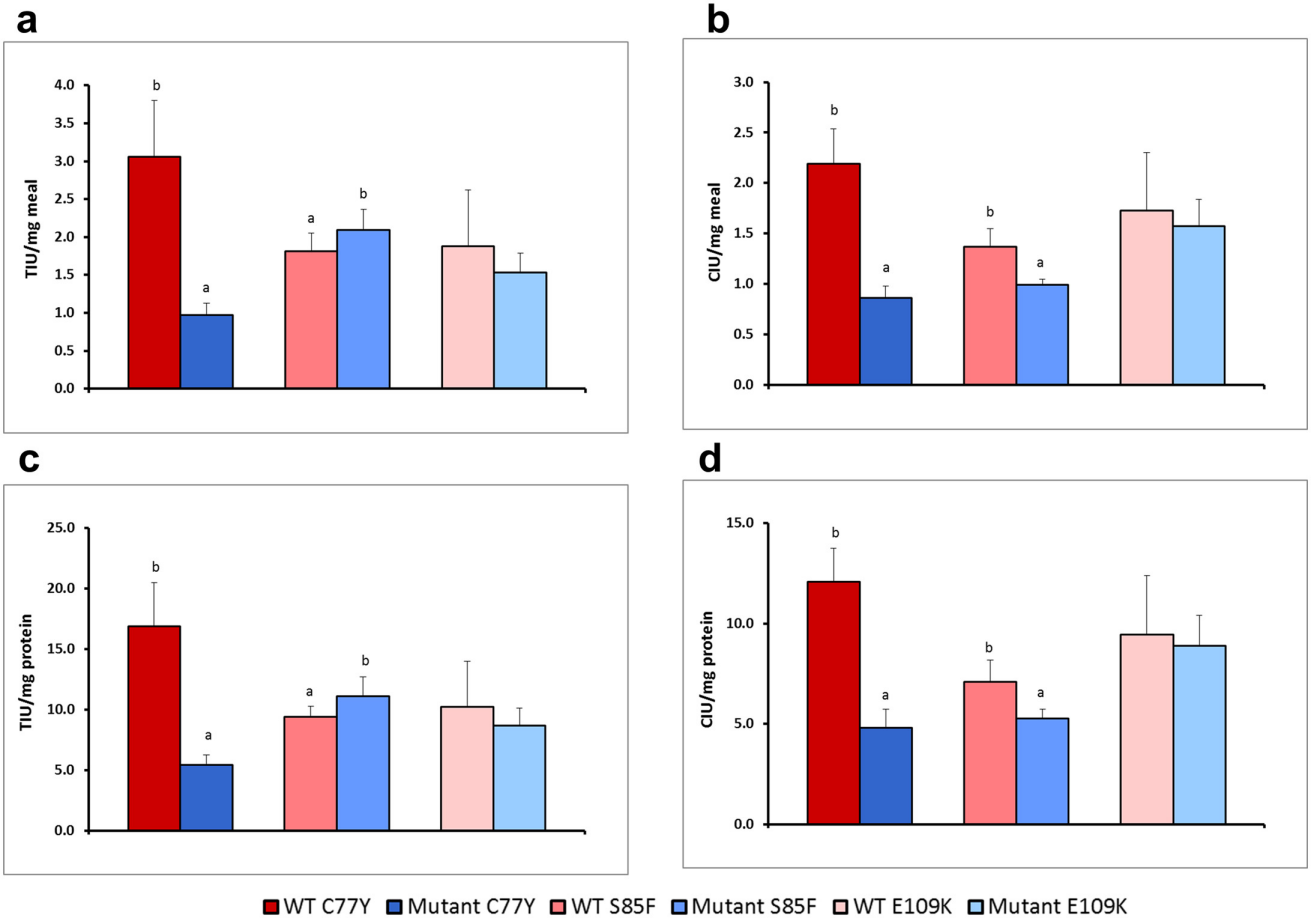
Impact of mutations on enzyme inhibition. Trypsin (TIU, **a, c**) and chymotrypsin (CIU, **b, d**) inhibitory units per mg of meal (**a, b**) or per mg of protein (**c, d**) of three TILLING mutants (C77Y, S85F, E109K) and their corresponding wild-type pea lines. For each plot, significant differences (p < 0.01) between wild-type and mutant lines within each pair are denoted (a, b, as appropriate on bars in each chart).

Measurement of overall trypsin and chymotrypsin inhibitory activities of seed protein extracts revealed a number of significant differences between mutant and wild-type lines (Fig 2). For the C77Y family, a significant reduction of greater than 60% was apparent for both trypsin (TIA) and chymotrypsin inhibitory activity (CIA) in mutant compared with wild-type lines. For the S85F family, a small but significant increase in TIA and a decrease in CIA were apparent in mutant compared with wild-type lines. For the E109K family, a slight but not significant decrease in TIA and CIA was apparent in mutant compared with wild-type lines. The same trends were observed for mutant compared with wild-type lines for TIA (Fig 2a and 2c), CIA (Fig 2b and 2d) and when expressed on a seed meal (Fig 2a and 2b) or seed protein (Fig 2c and 2d) basis.

**Fig 3:**
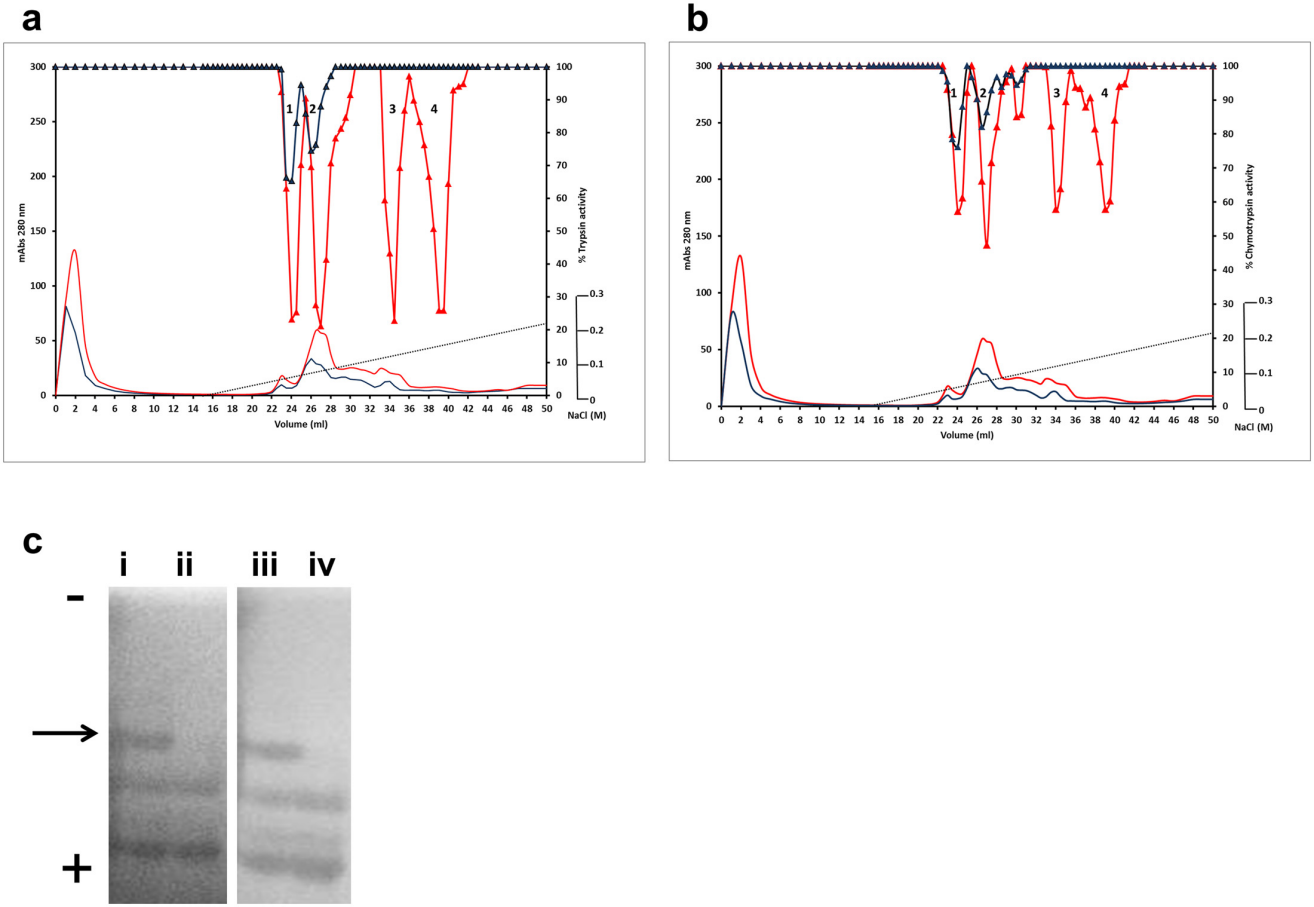
Enzyme inhibitory profile of the TI1 C77Y mutation. Seed proteins from wild-type and mutant lines from the TI1 C77Y family were separated by cation-exchange chromatography. Absorbance profiles at 280 nm are shown (**a, b**: red and blue lines without symbols; mAbs, left-hand scale). Using BAPNA and BTEE as specific substrates, the trypsin (**a**) and chymotrypsin inhibitory (**b**) activities of wild-type (red triangle) and mutant (blue triangl) protein fractions are shown, relative to assays where trypsin activity is 100% (control; right-hand scale). Numbers in peaks in each chromatogram correspond to the different forms of TI1 and TI2 (peak 1: TI2 processed; Peak 2: TI2 unprocessed; Peak 3: TI1 processed; Peak 4: TI1 unprocessed. (**c**) in-gel protease inhibitory activity of inhibitor isoforms from wild-type (i, iii) and C77Y mutant (ii, iv) lines. Zymogram blue casein gels were treated with the digestive enzymes, trypsin (i, ii) or chymotrypsin (iii, iv); dark areas indicate where the enzyme has been inhibited. The direction of electrophoresis on non-denaturing gels is indicated (-, +). The arrow indicates the position of the isoform that is missing from the mutant lines (tracks ii and iv).

**Fig 4:**
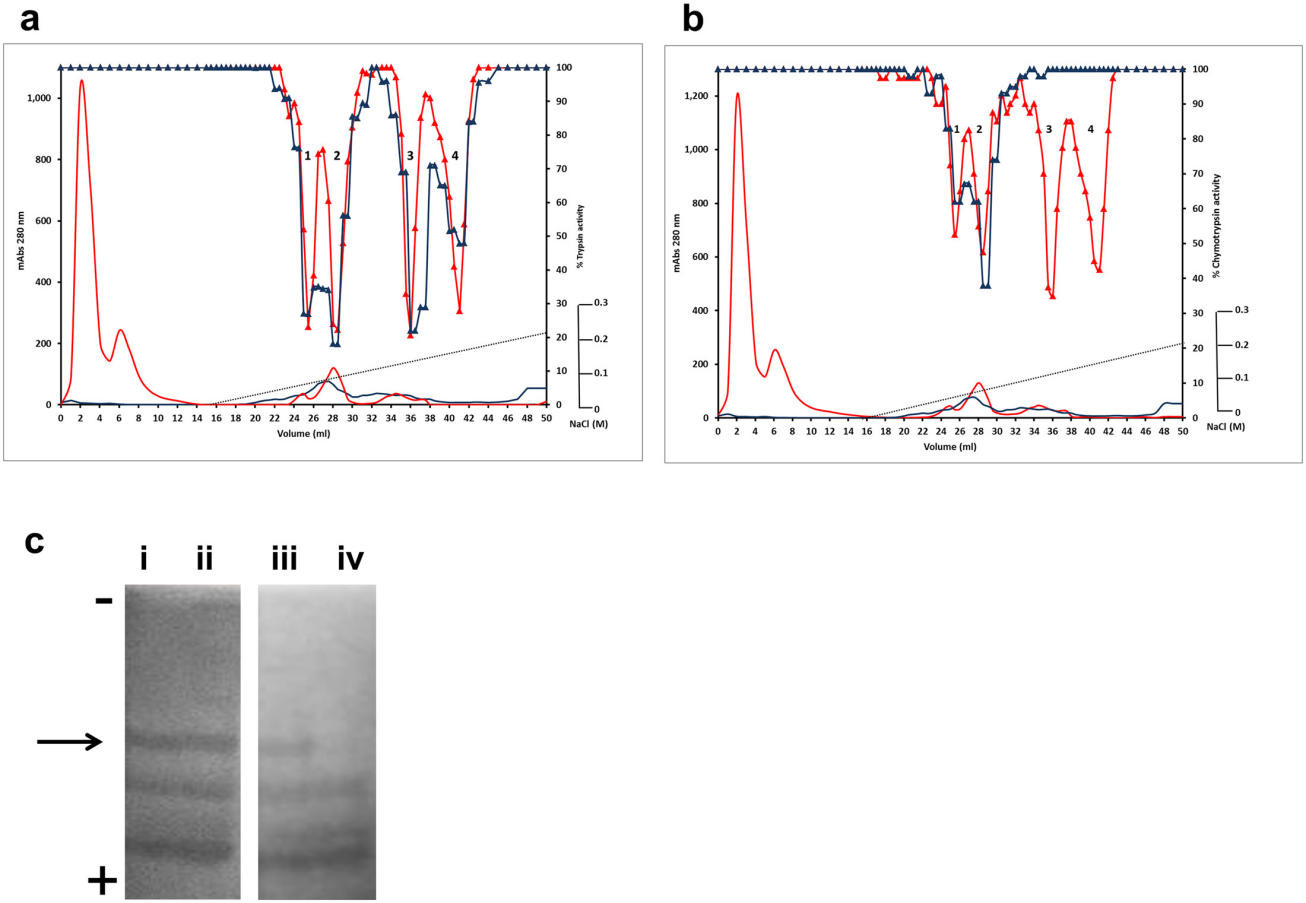
Enzyme inhibitory profile of the TI1 S85F mutation. Seed proteins from wild-type and mutant lines from the TI1 S85F family were separated by cation-exchange chromatography. Absorbance profiles at 280 nm are shown (**a, b**: red and blue lines without symbols; mAbs, left-hand scale). Using BAPNA and BTEE as specific substrates, the trypsin (**a**) and chymotrypsin inhibitory (**b**) activities of wild-type (red triangle) and mutant (blue triangle) protein fractions are shown, relative to assays where trypsin activity is 100% (control; right-hand scale). Numbers in peaks in each chromatogram correspond to the different forms of TI1 and TI2 (peak 1: TI2 processed; Peak 2: TI2 unprocessed; Peak 3: TI1 processed; Peak 4: TI1 unprocessed. (**c**) in-gel protease inhibitory activity of inhibitor isoforms from wild-type (i, iii) and S85F mutant (ii, iv) lines. Zymogram blue casein gels were treated with the digestive enzymes, trypsin (i, ii) or chymotrypsin (iii, iv); dark areas indicate where the enzyme has been inhibited. The direction of electrophoresis on non-denaturing gels is indicated (-, +). The arrow indicates the position of the isoform that is missing from the mutant lines (track iv).

**Fig 5:**
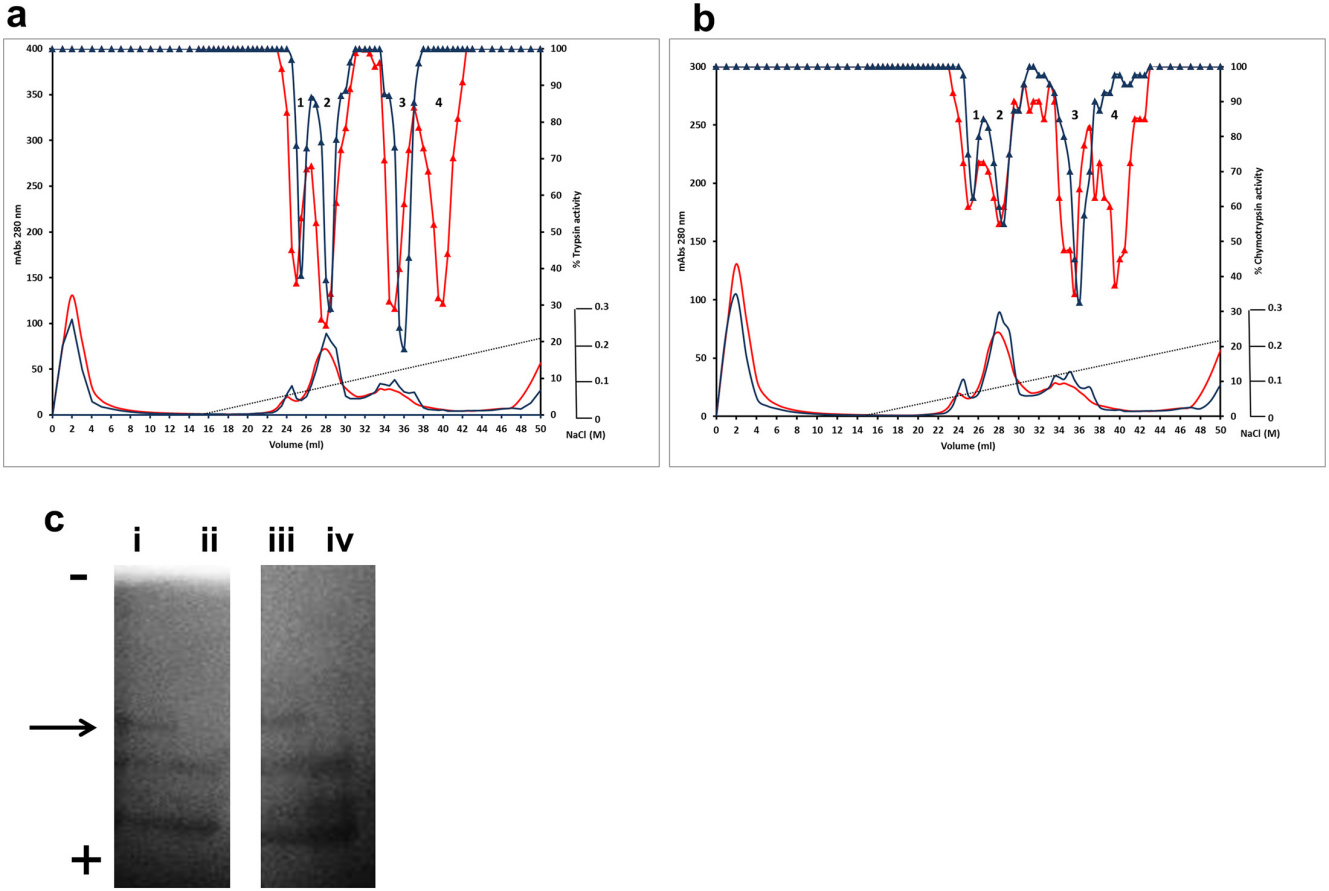
Enzyme inhibitory profile of the TI1 E109K mutation. Seed proteins from wild-type and mutant lines from the TI1 E109K family were separated by cation-exchange chromatography. Absorbance profiles at 280 nm are shown (a, b: red and blue lines without symbols; mAbs, left-hand scale). Using BAPNA and BTEE as specific substrates, the trypsin (**a**) and chymotrypsin inhibitory (**b**) activities of wild-type (red triangle) and mutant (blue triangle) protein fractions are shown, relative to assays where trypsin activity is 100% (control; right-hand scale). Numbers in peaks in each chromatogram correspond to the different forms of TI1 and TI2 (peak 1: TI2 processed; Peak 2: TI2 unprocessed; Peak 3: TI1 processed; Peak 4: TI1 unprocessed. (**c**) in-gel protease inhibitory activity of inhibitor isoforms from wild-type (i, iii) and E109K mutant (ii, iv) lines. Zymogram blue casein gels were treated with the digestive enzymes, trypsin (i, ii) or chymotrypsin (iii, iv); dark areas indicate where the enzyme has been inhibited. The direction of electrophoresis on non-denaturing gels is indicated (-, +). The arrow indicates the position of the isoform that is missing from the mutant lines (tracks ii and iv).

The differences in inhibitory activity among mutants were investigated further following fractionation of the different isoforms corresponding to the closely related *TI1* and *TI2* genes in pea [12,27]. The major isoforms in seeds have been shown to correspond to mature and carboxy-terminally processed forms for each gene product [11]. Figs 3–5 show the profiles of total protein extracts from mutant and wild-type families (C77Y, S85F and E109K, respectively), when seed proteins are separated by cation-exchange chromatography and assayed for their ability to inhibit trypsin and chymotrypsin. Four isoforms were apparent among the separated seed proteins from all wild-type control lines (Figs 3a–5a and 3b–5b, upper red traces); the four isoforms were evident as fractionated protein peaks (labelled 1–4) with the ability to inhibit both trypsin and chymotrypsin. Peptides from each of these four peaks were identified in seeds from wild-type families and the parent cultivar (cv.) Cameor as mature and processed products of the *TI1* and *TI2* pea genes (Table 2). Peaks 1 and 2 contain the TI2 protein, with a diagnostic D residue at the P2’ position of the trypsin inhibitory domain in the deduced sequences. Peaks 3 and 4 contain the TI1 protein, with diagnostic N residues at the P2’ position of both inhibitory domains as well as Y and K residues at P1 and P5’ of the chymotrypsin inhibitory domains among deduced sequences. The presence of the TI1 and TI2 carboxy-terminal motif, CHNSEVEEVIKN, in peptides from peaks 2 and 4 indicates that these peaks contained the mature unprocessed TI2 and TI1 proteins, respectively (Table 2). The determined carboxy-terminal sequence includes the nonapeptide previously shown to be removed from a sub-set of the primary mature proteins in *vivo* [11,27]. We conclude, therefore, that the order of elution (Figs 3–5) is: TI2 processed, TI2 unprocessed, TI1 processed and TI1 unprocessed, at variance with predicted charges within each class (+4, +3, +6.5 and +6, respectively, at pH 4).

**Table 2:** Identification of TI1 and TI2 diagnostic peptides by mass spectrometry.

Peptide	* *
**TI2**	GDDVKSACCDTCL CTKS**D**PPTC RCVDV**G**ETCHSACDSCI CA**L**S**Y**PP**Q**C QCFDTHKFCYKACHNSEVEEVIKN
**TI2** (P1)	GDDVKSACCDTCL CTKS**D**PPTCR
**TI2** (P2)	GDDVKSACCDTCL CTKS**D**PPTC R——————————————————ACHNSEVEEVIKN
**TI1**	GDDVKSACCDTCL CTKS**N**PPTC RCVDV **R** ETCHSACDSCI CA**Y**S**N**PP**K**C QCFDTHKFCYKACHNSEVEEVIKN
**TI1** (P3)	——-SACCDTCL CTKS**N**PPTC RCVDV **R** ETCHSACDSCI CA**Y**S**N**PP**K**
**TI1** (P4)	——-SACCDTCL CTKS**N**PPTC RCVDV **R** ETCHSACDSCI CA**Y**S**N**PP**K**C QCFDTHKFCYKACHNSEVEEVIKN

Identification of TI proteins eluted by cation-exchange chromatography (P, peaks 1–4) of wild-type seed extracts, as shown in Figs 3–5. The peptides obtained for P1 and P2 corresponded to the amino acid sequence for TI2 (UniProt accession: Q41066), whereas those deduced for P3 and P4 corresponded to the amino acid sequence for TI1 (UniProt accession: Q41065). The sequences of the inhibitory domains are underlined. The amino acid residues that distinguish TI1 and TI2 proteins are shown in bold. Lys (K) and Leu (L) or Tyr (Y) at position P1 (*) determine specificity for trypsin and chymotrypsin, respectively.

In contrast to the four isoforms distinguished in the wild-type inhibitor profiles, only two isoforms were evident among fractionated seed proteins from the C77Y mutant which showed inhibition of both target enzymes (Fig 3a and 3b). These data suggest that the two isoforms which are derived from the *TI1* gene, and which elute latest from the cation-exchange column separation of the wild-type inhibitors (peaks 3 and 4), show no activity in the C77Y mutant. Analysis of seed protein extracts on native gels that are stained for TIA and CIA (Fig 3c) supports the loss of one of three inhibitor isoforms from the C77Y mutant; the TI2 isoforms common to both wild-type and mutant lines are more electronegative under the electrophoresis conditions used. Under these conditions, the carboxy-terminally processed product of the *TI1* gene is predicted to be uncharged and is not detected on the activity gels of wild-type seed extracts. Overall the loss of inhibitory activity associated with two TI1 isoforms is in agreement with the C77Y mutation leading to a loss of inhibitor function at the two protein domains. The behaviour of TI1 and TI2 isoforms on cation-exchange columns and non-denaturing gels at pH 4.4 and pH 7.0, respectively, in the mutant is in agreement with the predicted charges of the two classes of proteins, where TI1 isoforms are more positively charged than those corresponding to TI2. The reduction of more than 60% in both TIA and CIA (Fig 2) in the C77Y mutant implies a greater contribution of TI1 to overall TIA. This could be because TI1 is a more potent inhibitor or because TI1 represents a greater proportion of total TI seed proteins. The first possibility may be supported by studies of the two individual pea seed inhibitors expressed in a heterologous system [29]; qPCR analyses were carried out to investigate the second possibility. The latter revealed that, although TI2 was expressed more highly in early stages of seed development (C5, C6, C8 stages), both genes were equally expressed later in development (C9, C10 stages) when the bulk of the TI proteins were synthesised (S2a and S2b Fig). Genomic DNA amplifications using gene-specific primers in forward and reverse combinations gave rise to an amplicon of >10 kb in two pea lines (cv. Cameor and JI1294), using primers designed on the *TI1* and *TI2* genes (sense strand), indicating a tail-to-tail orientation of the two genes (S2c Fig). This gene arrangement with more remote promoter regions than in a tandem array may provide an explanation for the marginally earlier expression of one gene compared with the other, as noted by qPCR analysis for *TI2*. Overall, however, there was no evidence that the *TI1* gene was expressed at a significantly higher level than *TI2*; based on this and earlier data [29], the loss of more than 60% TIA and CIA in the C77Y mutant likely reflects differences in the respective inhibitory activities of TI1 and TI2.

Four isoforms were apparent when seed proteins were separated from wild-type segregant lines corresponding to the S85F mutant family (Fig 4a and 4b). In contrast, among fractionated seed proteins from the S85F mutant lines, four isoforms showed inhibition of trypsin (Fig 4a) but the chymotrypsin inhibitory activity of peaks 3 and 4, corresponding to TI1 isoforms, was completely abolished (Fig 4b). Analysis of seed protein extracts on native gels that are stained for TIA showed no difference in isoform pattern between wild-type and mutant lines but a loss of CIA was evident for one of three inhibitor isoforms in the S85F mutant lines (Fig 4c); this one corresponds to the TI1 isoform that is evident on gels of wild-type lines. Its apparent loss in the S85F mutant is consistent with abolition of CIA as a consequence of loss of the active site serine residue; TIA is not affected negatively by this mutation. The decrease in overall CIA in the S85F mutant compared with the control line was expected to be comparable to that observed for the C77Y mutant (Fig 2b); however the decrease was lower than expected, likely due to the lower overall activity in the wild-type line, compared with other controls. This may be explained by the mutant and corresponding wild-type lines being BC2; differences between control lines in the different mutant families would be expected to diminish with further backcrossing.

Fig 5 shows a similar analysis of the E109K mutation. This third TILLING mutation lies within the carboxy-terminal region that is removed from the processed TI1 isoform, and so should not impact directly on its ability to inhibit target proteases. Since the E109K mutation leads to a change in overall charge of the unprocessed mutant protein, the inhibitor profile was expected to differ in the case of the mutant protein irrespective of any associated changes in activity. The predicted change (more positive charge) is in agreement with the apparent loss of activity that is associated with the last eluting inhibitor (peak 4) observed for wild-type lines, corresponding to unprocessed TI1 (Fig 5a and 5b). Given that no additional or later chromatographic peak having protease inhibitory activity was found in the mutant protein, it is likely that both forms of the TI1 protein co-eluted in peak 3 (see above regarding variance of actual vs. predicted charges). Analysis of seed protein extracts from the E109K mutant and corresponding wild-type lines on native gels (Fig 5c) confirms the apparent loss of the unprocessed TI1 protein due to the change in overall charge. Here both processed and unprocessed TI1 would be expected to be uncharged at pH 7.0.

**Fig 6:**
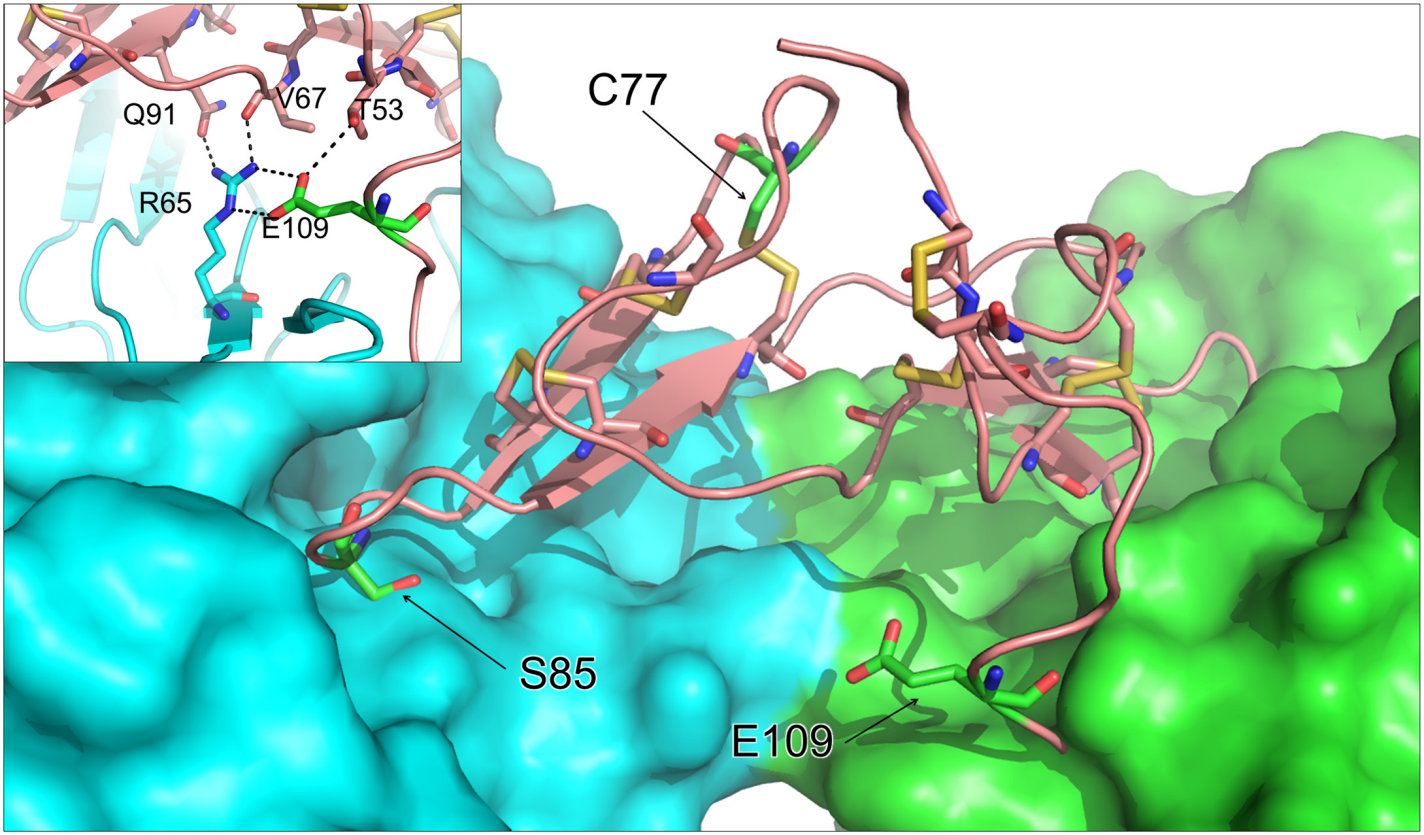
Structural impact of mutations. Homology model of pea TI1 (based on PDB entry 1PBI) is shown as a pink ribbon bound to bovine trypsin, shown as blue and green molecular surfaces (created by superposing the pea TI1 model onto the structure of the ternary complex of *Medicago scutellata* BBI (not shown) bound to bovine trypsin taken from PDB entry 2ILN). The positions of the three induced mutations are indicated and shown with green carbon atoms. **Inset:** The extended hydrogen-bonding network likely to be important in dimer formation, based on two copies of the pea TI1 model superposed onto the dimer of the template structure (PDB entry 1PBI), is indicated by dashed lines.

The impact of the mutations on the likely interaction between protease inhibitors and target enzymes was studied in terms of protein structure. Fig 6 shows the model of the wild-type TI1 in complex with trypsin, where the positions of the three mutations studied here are shown. The C77Y mutation, despite not being involved directly in the inhibitory domains, leads to a loss of one of the seven highly conserved disulphide bridges (C77-C92; Fig 1b), and may be predicted from the model to lead to a loss of structural rigidity. In particular, this could adversely affect the presentation of the chymotrypsin inhibitory loop and therefore its efficacy as a substrate mimic. The S85F mutation affects the P1’ position of the inhibitory site that engages directly with the chymotrypsin active site and the substitution introduces a bulky aromatic side chain that would be predicted from the model to abrogate binding (as supported by the absence of CIA in TI1 peaks; Fig 4b). In the case of the E109K, this region of the structure is not visible in any of the complexes that are available in databases (PDB entries 2ILN, 3RU4, 3MYW, 1D6R, 1TAB and 2G81), suggesting that it is flexible or cleaved and plays no significant role in the interaction between protease inhibitor and target enzyme. The position of E109 in Fig 6 is based on the structure of the free homodimeric inhibitor (PDB entry 1PBI). However, it seems likely that E109 may be important in dimer formation, via an extended hydrogen-bonding network that would be important in such interactions (see inset to Fig 6). Although the E109K substitution may not disrupt these interactions, it could result in a different or disordered conformation for the carboxy-terminus and an overall weaker dimer interface. The mutation could therefore impact on the overall equilibrium among TI1 monomers, dimers and enzyme bound isoforms, whether processed or unprocessed; however the activities measured for E109K mutant and wild-type lines do not suggest that any such impact will have major consequence for overall activity (Fig 2), at least under the assay conditions used.

### Oligomerization pattern of the mutant E109K

**Fig 7:**
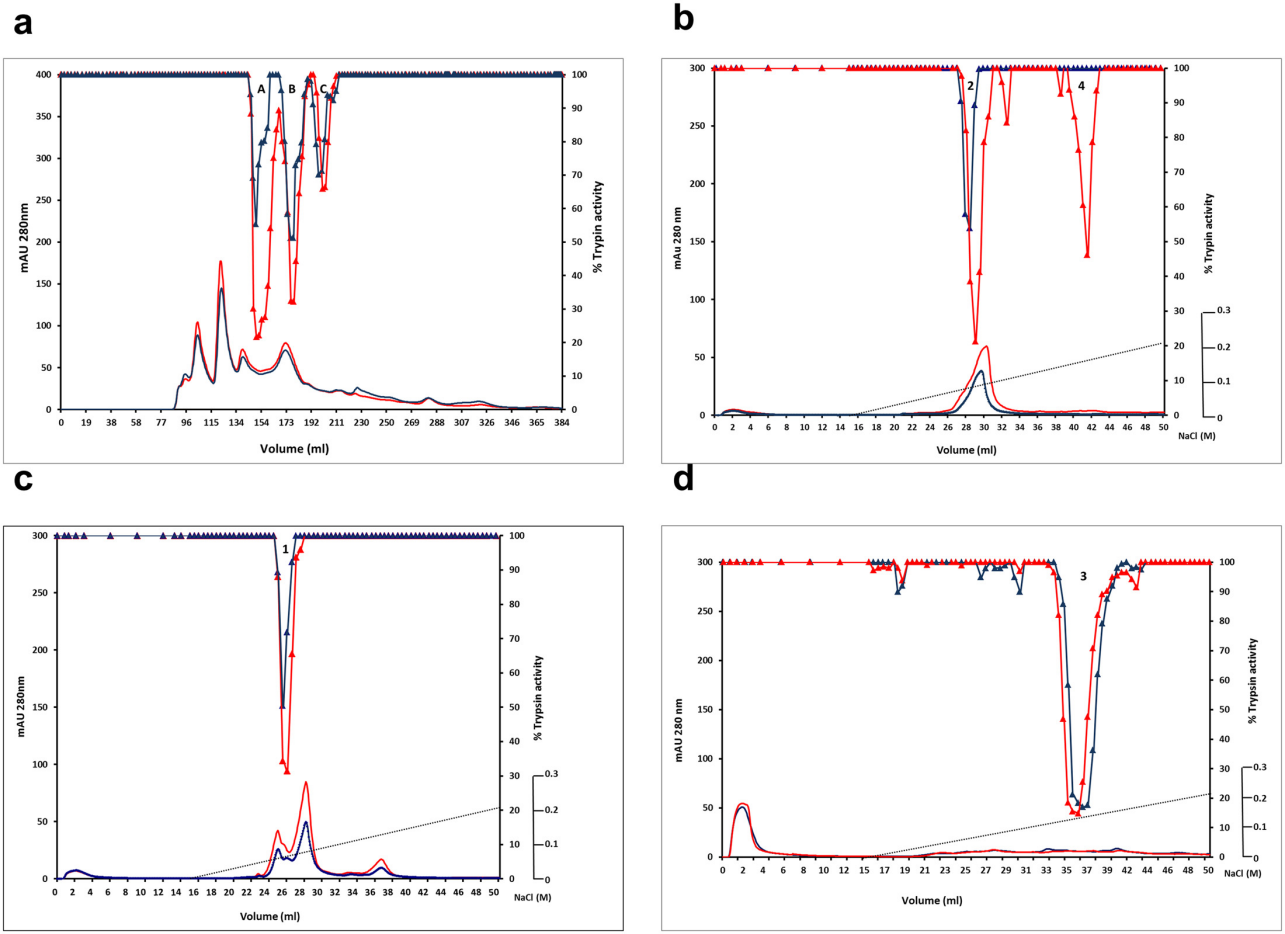
Oligomerization status of TI isoforms in the E109K mutant compared with wild type. (**a**) Wild-type (red) and E109K mutant (blue) albumin preparations were analyzed by size-exclusion chromatography. Absorbance at 280 nm (left-hand scale) is shown in lower traces; inhibition of trypsin activity in fractions from wild type (red) and E109K mutant (blue) are shown in upper traces, where the % residual trypsin activity is indicated on the right-hand scale. (**b-d**) Cation-exchange chromatography of the size-excluded peaks A, B and C, respectively, from wild type (red) and E109K mutant (blue) (see a). For each analysis, absorbance at 280 nm (left-hand scale) is shown in lower traces; inhibition of trypsin activity in fractions from wild type (red) and E109K mutant (blue) are shown in upper traces, where the % residual trypsin activity is indicated on right-hand scale. Numbers in peaks in each chromatogram correspond to the different forms of TI1 and TI2 (peak 1: TI2 processed; Peak 2: TI2 unprocessed; Peak 3: TI1 processed; Peak 4: TI1 unprocessed.

The possible effect of the E109K mutation on the oligomerization pattern of TI1 and TI2 isoforms was investigated by size-exclusion chromatography. Under the conditions employed, a linear logarithmic response for elution of five standard proteins in the range 6,500 to 63,500 molecular weight was observed (R² = 0.974; S3 Fig). Analysis of albumin extracts from cv. Cameor (not shown), wild-type control and E109K mutant lines by size-exclusion chromatography showed three chromatographic peaks (named A, B and C) containing TIA (Fig 7a). Interestingly, the relative peak areas for TIA differed appreciably between the E109K mutant and wild-type control lines; in particular, the activity of peak A was significantly higher in the wild type than in the E109K mutant. This indicated that protein from the wild type showed a higher relative abundance of the oligomeric TI forms, deduced to be dimers [28,30], when compared with the E109K mutant. The composition of the three oligomeric TI forms was investigated by cation-exchange chromatography where, as shown earlier, four and three isoforms could be resolved for wild-type and E109K mutant lines, respectively (Fig 5a and 5b). In the wild-type lines, the size- excluded peak A was shown to be composed of unprocessed TI2 and TI1 proteins, whereas peaks B and C contained carboxy-terminally processed TI2 and TI1, respectively (peak numbers 2 and 4, 1 and 3, respectively, in Fig 7b–7d). In contrast, in the E109K mutant, the size-excluded peak A was shown to be composed of unprocessed TI2 protein only whereas, in agreement with analysis of the wild-type protein, size-excluded peaks B and C contained carboxy-terminally processed TI2 and TI1, respectively. In the E109K mutant, the unprocessed TI1 showed altered behaviour on cation-exchange chromatography due to the mutation (see earlier), so it might be concluded that both TI1 isoforms are present in the size-excluded peak C from the mutant.

The combined data suggest a reduction in the degree and type of oligomers that are formed from TI1 in the E109K mutant compared with wild type. The highest molecular weight form (Peak A) was reduced in relative amount and in complexity in the mutant, indicating strongly that the carboxy-terminus influences the extent to which dimers are formed and that the charge difference in the E109K mutant interferes with this process.

### Identification of natural *TI* mutants

**Fig 8:**
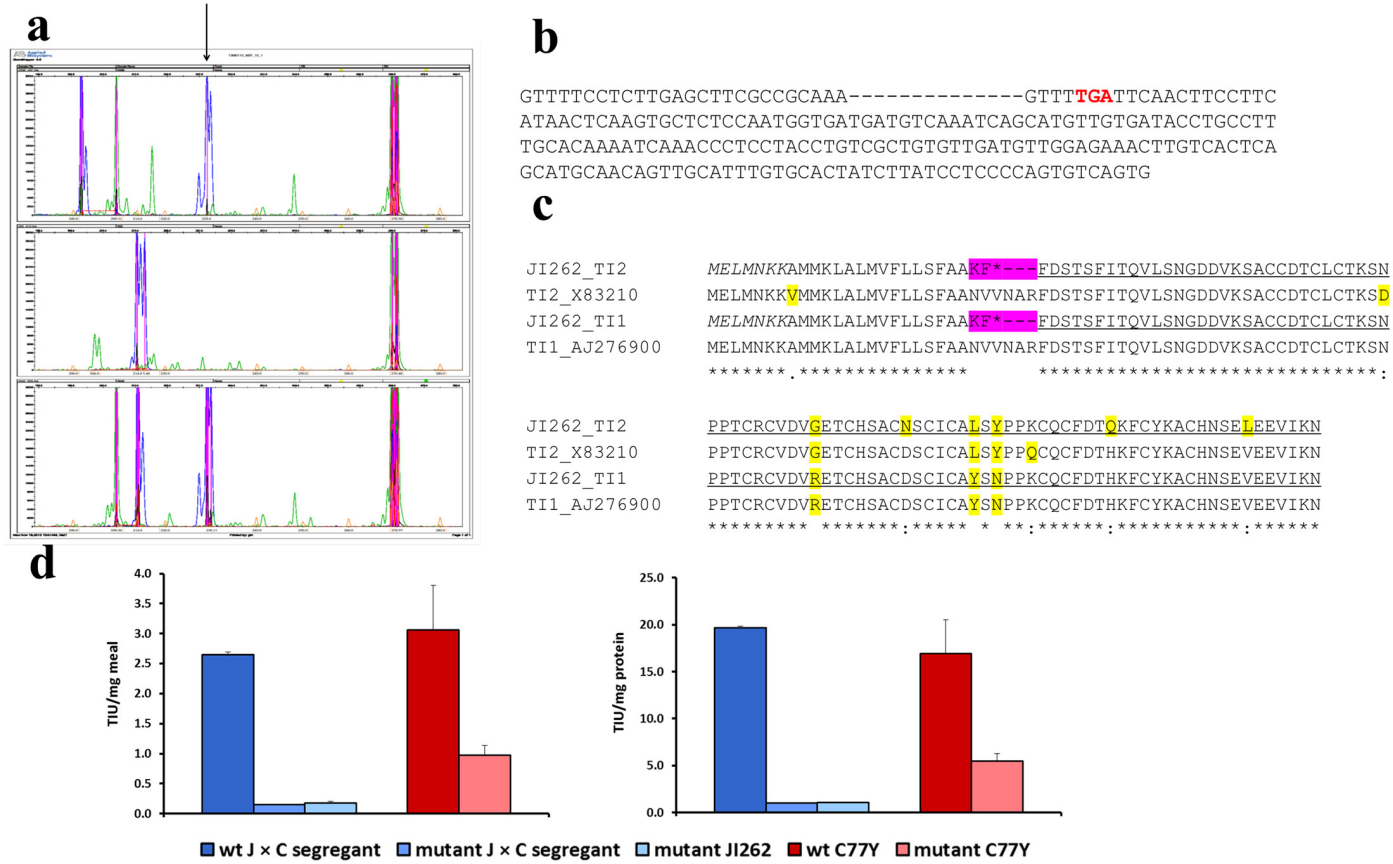
Identification of a *TI1/TI2* double null mutant in a pea germplasm collection. (**a**) Multiplex screening of pea germplasm DNA for seed protein gene variants. The predicted *TI2* amplicon evident in top and lower panels is highlighted with an arrow; this amplicon is absent from the line analysed in the middle panel: JI 262. (**b**) *TI2* sequence amplified from JI 262. The 14 bases missing are indicated with dashes, leading to a premature stop codon (red font). (**c**) Predicted amino acid sequence of the mutant JI 262 genes, compared with wild-type TI1 and TI2; the premature stop codons (*) occur within the 42 amino acid pre-pro-peptide (region affected by the mutation highlighted pink). Primer-encoded amino acids in italics; predicted amino acid sequences beyond deletions underlined; variant amino acids highlighted. (**d**) TIA of the mutant, JI 262, and a mutant F2 segregant compared with a wild-type F2 segregant from a cross between JI 262 and cv. Cameor (J x C). For comparison, the C77Y mutant and control lines (Fig 2) are plotted alongside. TIU per unit meal (left), per unit protein (right).

In parallel with the isolation and analysis of induced TI mutants, natural germplasm variants were sought by performing a fluorescent multiplex genetic marker screen. The multiplex screen of *Pisum* germplasm DNA led to the identification of lines showing a loss of some of the expected fluorescently-labelled amplicons for a number of seed protein genes. Since loss of an amplicon could reflect divergence of primer sites, rather than a deletion of a target gene or part of the gene, all variants were re-tested using the same and alternative outer primer pairs in single PCR; the alternative primer pair spanned the region covered by the multiplex screen. In the case of the targeted *TI* gene (*TI2)* amplicon, one variant was identified which lacked the expected 230 bp amplicon in the multiplex screen (Fig 8a). Further analysis of this variant (JI 262, a *Pisum elatius* accession) revealed that the *TI2* amplicon was 14 bp shorter than that of wild type (Fig 8b), predicting a TI protein which terminates early as a consequence of the deletion and consequent loss of reading frame within the pre-pro-peptide (Fig 8c). The variant was predicted to lack TI2.

Since measurements of protease inhibitory activity indicated a very extreme reduction in TIA and CIA in the natural variant, JI 262, much higher than expected for loss of *TI2* gene function alone (see below), analysis was carried out on *TI1* gene structure in JI 262. Using forward primers designed on the 14bp deleted region of *TI2*, together with the *TI1* and *TI2* diagnostic reverse primers (At^YSN^ and At^LSY^, respectively), yielded no amplicon from JI 262 but the expected two from the wild type, cv. Cameor. Further analysis of four independent plants of JI 262 and a F1 plant (JI 262 x Cameor) using the primer combinations above, or using an alternative forward primer designed on the conserved amino terminus of the proteins, indicated that both *TI1* and *TI2* genes in JI 262 have the same deletion (Fig 8c). Analysis of F1 (JI 262 x Cameor) plants using the forward primer based on the 14 bp deletion yielded a product that was identical to that of cv. Cameor with both *TI1* and *TI2* reverse primers, supporting the lack of amplification of either the JI 262 *TI1* or *TI2* allele in the F1. The F1 hybrid status was clear using primers that amplified outside of the deletion for either gene (not shown).

The TIA and CIA determined for seeds of JI 262 suggested that the overall inhibitory activity was significantly and very severely reduced (0.15–0.2 TIU/mg flour, with CIA being undetectable), compared with cv. Cameor and other pea control samples. The extent of reduction was investigated further in the cross derived from JI 262 with cv. Cameor and by analyzing segregants having mutant or wild-type *TI* alleles. Fig 8d shows that F2 segregants with the mutant *TI* alleles had very low TIA, comparable to that of JI 262. Furthermore, mixing equal amounts of seed meals from a mutant segregant and cv. Cameor reduced the TIA of the latter by 50% (data not shown). In combination, these data are consistent with the low TIA of JI 262 being a consequence of *TI* genetic variation, rather than an additional seed component acting as an inhibitor of TIA. In JI 262, TIA is reduced at least 15-20-fold, compared with wild-type controls; for comparison, the TIA determined for the TILLING C77Y and wild-type controls are shown in Fig 8d.

The data presented above show unequivocally that a null mutant for the major pea seed protease inhibitors has been identified as a single accession within a diverse *Pisum* collection, as a consequence of a deletion within both the *TI1* and *TI2* genes.

## Discussion

In this work, we describe the use of TILLING and germplasm resources to identify and characterise mutations which provide novel opportunities for understanding functional aspects of seed proteins and provide for seed quality improvement. The availability of a TILLING platform in pea has accelerated greatly the ability to carry out functional genomics in this important cool season legume crop. Trypsin-chymotrypsin inhibitor gene families have been a focus for seed improvement strategies in many legume crops and some successes noted for reducing their activity in soybean [16,17,19–22]. In pea, although quantitative variants had been identified and the molecular basis of variation in gene expression described for the major seed inhibitor class [31], null or other extreme variants had not been identified or generated hitherto. Here we describe and characterise four (three induced, one natural) mutations that provide novel allelic variation for *TI* genes in pea; of the induced mutations, two were associated with a dramatic reduction in inhibition of one or both of the target enzymes, trypsin and chymotrypsin, whereas a third impacted on the extent to which TI oligomers are formed. A naturally occurring TI1/TI2 double null variant was identified and shown to have extremely reduced TIA and undetectable CIA in its seeds; this is a wild *Pisum* species (*Pisum elatius*) which has been crossed readily with cultivated *Pisum sativum*. The extremely low TIA (and no CIA) measured in seeds of this mutant is likely attributable to those genes that are expressed predominantly in vegetative organs of pea plants, and which are barely active in seed organs; these genes encode proteins that have two trypsin inhibitory domains and lack a chymotrypsin inhibitory domain [12].

The three induced mutant *TI1* genes described here enabled studies of the impact of i) loss of one of the 14 cysteine residues, which are highly conserved within proteins of this class (C77Y mutation, where C77 is predicted to participate in a disulphide bond close to the chymotrypsin inhibitory loop), ii) loss of the active site serine within the chymotrypsin inhibitory loop (S85F) and iii) altered charge within the carboxy-terminal domain (E109K) (Fig 1). The mutant lines were back-crossed twice to cv. Cameor to give BC2F2-derived mutant lines, ensuring that the effects of background mutations were reduced in the further analysis of the effects of individual mutations.

The results show the dramatic loss of both TIA and CIA associated with TI1 in the C77Y mutant, when TI1 and TI2 isoforms were fractionated from mutant and corresponding wild-type lines (Fig 3). These data provide unequivocal evidence that, of the seven disulphide bonds predicted to stabilise the activity loops of double-headed inhibitors, at least that involving C77 is absolutely critical for overall inhibitory activity. The overall activity of the C77Y mutant was reduced by more than 60% compared with wild type (Fig 2), indicating that the second major seed inhibitor, TI2, contributes less to overall activity than TI1. These data are in agreement with earlier studies of recombinant proteins representing TI1 and TI2, where TI1 isoforms showed greater inhibition of chymotrypsin than did TI2, hypothesised to reflect the active site sequences (AYSN in TI1 compared with ALSY in TI2), in agreement with tyrosine being a more effective amino acid at the P1’ position [29,32]. As far as trypsin inhibition is concerned, there is some evidence to suggest that TI2 may also be less effective than TI1 (TKSN in TI1, compared with TKSD in TI2) [33], in agreement with the lower activity remaining in the C77Y (TI1) mutant (Fig 2). It is also possible, however, that dimers of TI1 and TI2 contribute significantly to activity and that a loss of functional TI1 leads to a disproportionate loss of overall activity; this theory is not supported by analysis of the E109K mutant, where a reduction in the extent to which oligomers are formed did not diminish TIA or CIA significantly. The expression of *TI1* and *TI2* genes was approximately equal during seed development at stages when protein is being maximally synthesised; the slightly earlier expression of *TI2* observed during seed development might reflect the genomic organisation of the two genes, which may be concluded not to influence their relative expression to any great degree (S2c Fig). In *Phaseolus*, it has been shown that two *TI* genes exist in a head-to-head arrangement [34] but this does not appear to hold for either pea (tail-to-tail; S2c Fig) or *Medicago truncatula* (see http://jcvi.org/cgi-bin/medicago/manatee/genelist_display.cgi?db=mta4&user=access&password=access&file=results8509.orf&features=gene_name&ev_features=&at=gene_name&searchstr=Bowman for Bowman-Birk inhibitor genes (Medtr7g077160—Medtr7g077340), revealed by JBrowse as a tandem array on the *Medicago truncatula* chromosome 7, syntenic to pea linkage group V). The loss of the active site serine from the chymotrypsin inhibitory domain in the S85F mutant led to a total loss of CIA of the two TI1 fractionated isoforms (Fig 4b). In this case, TIA of the TI1 isoforms appeared to be unaffected (Fig 4a), although overall TIA was somewhat elevated (Fig 2a). The effect on TIA in the mutant is unexpected but may reflect structural changes in the protein overall as a consequence of the F85 residue, which could impact on steric effects in the binding of trypsin.

The E109K mutant showed marginal decreases in both TIA and CIA (Fig 2), consistent with the lack of direct participation of the carboxy-terminal domain in interactions with trypsin or chymotrypsin. Here, however, it was predicted that the charge variation might impact on interactions between monomers (Fig 6 inset). The data obtained from size-exclusion chromatography indicated that the E109K mutation had a profound effect on the participation of TI1 in oligomeric forms of TI. The composition of eluted peaks from size-exclusion chromatography of the E109K mutant (Fig 7) indicated that, whereas peak A from wild type consisted of unprocessed forms of TI1 and TI2, the TI1 unprocessed form was absent from peak A in the mutant E109K (Fig 7b), where peak A was reduced in proportion overall (Fig 7a). These data are supported by a suggested electrostatic interaction between the ε-amino group of K58 of one monomer and a negatively charged residue at the carboxy-terminal of the second monomer in the formation of dimers [35,36]. Although up to three glutamic acid residues are present in the carboxy-terminal tail of TI1 (Fig 1b), the residue E109 seems to be strictly necessary in the dimerization process of pea TI. Given the identical carboxy-terminal ends of TI1 and TI2 variants, the presence of heterodimers as well as homodimers in the wild type would be expected. The size-excluded chromatographic peaks B and C were shown to contain processed TI2 and TI1 isoforms (i.e. lacking the carboxy-terminal domain), respectively, and predicted to exist as monomers.

Monomeric and dimeric forms of pea TI are suggested to differ in shape, with the dimers being more compact [28]. In the present work, the monomeric processed TI1 and TI2 isoforms (size-excluded chromatographic peaks C and B) eluted with apparently different molecular weights in the range 12–15 and 22–30 kDa, respectively); differences in their amino acid sequences and the lack of compactness of the monomeric forms, mainly due to the presence of exposed hydrophobic surface patches, might explain this anomalous behaviour. TI dimerization results in the presence of four inhibitory domains located at the molecular surface. The reported crystal structure of PsTI-IVb from pea [28] and functional studies carried out on HGI-III from horsegram [36] suggest that the dimeric state of the molecule is more stable than the monomeric state; most of the exposed hydrophobic residues of the monomeric molecule are shielded from the solvent by the second subunit in the dimer. The functionality and stability of monomeric and dimeric TI forms in terms of protease inhibition in mutants may become relevant in interactions with enzymes other than those used here in standard assays, for example, enzymes such as matriptase [37] and the proteasome [38] implicated in anti-carcinogenic responses.

Targeted-screening of pea germplasm has proven the utility of this approach in identifying sources of novel germplasm that would be difficult to detect by other means. The variant, JI 262, a wild *Pisum elatius* line originating from Turkey, revealed a deletion that was common to both *TI1* and *TI2*, with a premature stop codon predicted for both proteins within the pre-pro-peptide region. The origin of such variation is intriguing, particularly as no other such variant was detected in the screening of 2822 *Pisum* lines. It is likely that the double mutation arose as a consequence of a gene conversion event involving the two related *TI* genes, one of which had acquired the original deletion. As a consequence, JI 262 showed an extreme reduction in TIA and no CIA. In this work, despite having extremely small seeds and a thick black testa, JI 262 has been readily crossed with a cultivar and mutant progeny lines used to show that the mutation is linked with low TIA. This variant may progress into breeding programmes, where progeny lines can be selected on the basis of phenotypes (short stature, lack of anthocyanins, round seeds), together with the molecular markers described here to follow the deletion. Beyond the opportunities for improved formulations for feed and food, and the higher inclusion of pea protein therein, the discovery of JI 262 opens possibilities for testing the function of TI proteins in seeds, and determining the extent to which these proteins contribute to plant and seed defence. The reductions in TIA/CIA achieved here for pea are higher than those obtained in soybean [21,22] and have the advantage of being conferred by a genetically linked pair of mutant alleles.

Studies in soybean have provided an alternative strategy for how seed protease inhibitor content may be manipulated. A soybean line expressing a mutant *BBI* transgene where both active sites have been interrupted with an inserted (glycine) residue showed a significant reduction in the amount of seed inhibitor; TIA was reduced from 20 to 50% in seeds of the transgenic soybeans [20]. The proposed explanation for the observed effects is that the more prevalent mRNA from the mutant gene, under the control of the phaseolin seed specific promoter, out-competed messenger RNA from the native genes during translation to decrease the amount of active BBI, possibly as a consequence of limiting sulphur amino acid content. Given the continuing lack of acceptance of transgenic technologies in Europe, the genetic improvement of seed quality by traditional mutagenesis and/or introgression of natural gene variants continue to be the most practical routes to breeding for improved feed and food.

The induced *TI1* mutant (C77Y) and the natural *TI* variant, JI 262, described in this study may be regarded as null mutants for one and two genes, respectively. The C77Y mutant retains TI2 function and offers a compromise in reducing TIA but retaining TIA for potential health-promoting properties [39,40]. As such, both mutants provide opportunities for the combination of mutations in order to reduce the content of anti-nutritional proteins in seeds. Null mutations have been reported for albumin 2 [41] and a lipoxygenase enzyme [42] previously in pea. More recently, several null mutations were identified following high-throughput screens of a population generated by fast neutron mutagenesis of pea [43]. Combinations of such mutations will provide an enhanced germplasm resource, predicted advantages in terms of protein quality, as well as novel variation to enable fundamental studies on the participation of seed protein gene families in indispensable plant functions that contribute to agronomic performance and ultimately yield.

This study demonstrates the potential for making major changes to the seed protein profiles of plant species, such that the demands for safe, high-quality, low allergenic protein sources can be met for an increasing world population as well as meeting the requirements of those with intolerance to cereal-based products.

## Materials and Methods

### Plant materials

The development of a mutagenized population as a TILLING resource in the pea cultivar (cv.) Cameor and its utilization (http://urgv.evry.inra.fr/UTILLdb), to isolate a number of allelic variants for several genes has been described [24,25]. M3 and M4 seeds from lines identified as carrying mutations in the *TI1* gene were sown in glasshouses; homozygous mutant lines were back-crossed twice to the cv. Cameor. BC2 F3 and F4 seeds from confirmed segregant mutant and corresponding wild-type lines were used for the preparation of seed meals. For every mutant line selected, one having the corresponding wild-type allele was selected from the same family as the control for that family.

Natural variants of *Pisum* germplasm, linked to a corresponding DNA resource based on single plants, are maintained at the John Innes Centre (JIC), Norwich, UK (http://www.jic.ac.uk/germplasm/). A variant line, JI 262, was crossed with cv. Cameor, and an F2 population developed to generate mutant and control segregants.

### Identification of *TI1* TILLING mutants

TILLING mutants for the trypsin/chymotrypsin inhibitor (TI) gene, *TI1*, were identified using primers based on the GenBank accession, AJ276900. Nested primers (N1, N2) were used to amplify the *TI1* gene, including promoter and downstream sequences. N1 primers (5’ GTAGCTTCATGCTATTGTTGCCT 3’, -371 to -349 relative to initiator ATG, and 5’ AAGTAATGActaaagtactatagatca 3’, 222 to 196 relative to terminator TGA) generated an amplicon of 938bp. N2 primers (5’ gcatggccttatgtctacagatgtgc 3’, -230 to -205 relative to initiator ATG and 5’ ttcacatgccacactgcacgatcatg 3', 137 to 112 relative to terminator TGA) generated an internal amplicon of 712bp. The generic screening methodologies for mutants among the TILLING population have been described [24].

Among a set of thirteen families with base changes in the *TI1* amplicon, three encoding missense mutations within the mature TI1 protein regions were selected for further study (Table 1). Mutant and wild-type lines were selected among BC2F2 segregants and their progeny confirmed using N1 and N2 primers (see above) in nested PCR analyses, followed by sequencing. BC2F3 and BC2F4 seeds were bulked for protein analyses and assays. All analyses and assays were based on two or three independent mutant and wild-type segregant lines for every family.

### Analysis of *TI1* gene expression and organisation

Quantitative PCR (qPCR) analysis was performed using RNA samples prepared from developing seeds of cv. Cameor at different stages of development. The control gene for qPCR experiments was *eIFα*, and qPCR conditions were as described [44,45]. Preliminary experiments were carried out to ensure that the dilutions of first strand cDNA used for qPCR were appropriate for comparisons and quantitative measurements of pea RNA for the target *TI1* and *TI2* genes. The primer pairs used to distinguish TI1 and TI2 cDNAs were: CTCTCCAATGGTGATGATGTC (QTI-CCf) and either TGACACTTGGGAGGATTAGAATA (At^YSN^) or TGACACTGGGGAGGATAAGATAG (At^LSY^) as reverse primer for *TI1* or *TI2*, respectively, where the At^YSN^ and At^LSY^ reverse primers are based on the distinct chymotrypsin inhibitory domains of the two proteins, respectively [31].

In order to ascertain the relative orientation of *TI1* and *TI2*, genomic PCR analysis was carried out under conditions appropriate to the generation of long products, using combinations of four primers: At^YSN^ and At^LSY^ primers (as above) and two primers complementary to At^YSN^ and At^LSY^ sequences [TATTCTAATCCTCCCAAGTGTCA (At5^YSN^-RC) and CTATCTTATCCTCCCCAGTGTCA (At5^LSY^-RC)]. PCR conditions were: 98°C for 1 min, (98°C for 10 sec, 55°C for 30 sec, 72°C for 7.5 min) x 35 cycles, and 72°C for 10 min, using ExTaq polymerase (Takara Bio Inc.), according to the manufacturer’s instructions. Alternative PCR conditions were: 98°C 1 min, (98°C for 10 sec, 55°C for 10 sec, 72°C for 5 min) x 36 cycles, and 72°C for 10 min, using I-proof polymerase (Bio-Rad Laboratories Inc.) according to the manufacturer’s instructions.

### Screening for natural *TI* mutants

DNA from 2822 *Pisum* accessions (maintained at the John Innes Centre (JIC), Norwich, UK; http://www.jic.ac.uk/germplasm/) as described [23], was used for the preparation of printed DNA plates for high-throughput targeted genetic screening. The DNA was prepared from individual plants, from which seeds were retained to form an independent resource (TG lines), linked to the marker database developed by Jing *et al*. [23]. This resource represents the broad genetic diversity available across the JIC *Pisum* accessions. Multiplex PCR assays were designed and carried out by iDna Genetics (http://www.idnagenetics.com/) to yield amplicons of 50–300 bp, based on a range of discrete gene—specific products that could be identified by fluorescent label and size. One primer of every pair carried a fluorescently labelled tag detector probe (either *Fam*, *blue* or *Vic*, *green*), to facilitate the detection of products, and additional bases (T) added to primers as necessary to distinguish gene products of otherwise similar predicted sizes. A multiplex screen was carried out to identify variants for a number of discrete genes; *TI* gene primers were: GTTTTCCTCTTGAGCTTCGCC and CACTGACACTGGGGAGGATAAGATAG (forward and reverse, nucleotides 88–108 and 317–292 of X83210, respectively, to yield a product of 230 bp). The details of the screening and detection of labelled amplicons were as described [43].

The variant, JI 262, was crossed with cv. Cameor, and F2 mutant and wild-type segregants identified by screening with *TI* gene-specific primers.

### Protein and gel analyses of TILLING mutants

Meals were prepared from wild-type and mutant seeds of replicate lineages for analysis of total protein profile on gels, total protein determination, analysis and quantification of albumins, measurement of trypsin and chymotrypsin inhibitory activities, fractionation of protease inhibitors by size-exclusion and cation-exchange chromatography and non-denaturing gel electrophoresis for zymography. Albumins were prepared, based on solubility in ammonium acetate as previously described [41] and total protein and fractionated proteins quantified using bovine serum albumin as standard.

Denaturing gel analyses were carried out using 12% or gradient 4–12% Bis-Tris pre-cast gels (Invitrogen), as described [43,46] and according to the manufacturer’s instructions, with 2-N-morpholine-ethane sulphonic acid (NuPAGE MES, Invitrogen) as running buffer. Immediately before loading, samples were reduced with DTT and NuPAGE antioxidant added to the upper buffer chamber to prevent re-oxidation of reduced proteins during electrophoresis. Gels were stained using InstantBlue (Expedeon, Harston, UK).

Non-denaturing gel separation of active protease inhibitor isoforms was carried out on 4–16% zymogram blue casein gels (Invitrogen) [47]. After electrophoresis, and following the manufacturer’s instructions, gels were treated with zymogram renaturating buffer (Invitrogen) for 30 min at room temperature, equilibrated with zymogram developing buffer (Invitrogen), incubated with 25 mL of trypsin or chymotrypsin solution (0.08 mg/mL of zymogram developing buffer) at 37°C for 1.5 h, and washed with distilled water before the addition of acetic acid to stop the enzymatic reaction.

### Measurement of protease inhibitory activities

Seeds were screened for their relative trypsin (TIA) and chymotrypsin inhibitory activity (CIA), as described previously [48]. Finely ground meal from 10–15 pooled seeds of every replicate pea line was used to measure TIA and CIA with *N*-α-benzoyl-DL-arginine-*p*-nitroanilide (BAPNA) [48] and *N*-α-benzoyl-L-tyrosine-*p*-nitroanilide (BTpNA) [49] as specific substrates, respectively. TIA and CIA, expressed as inhibitor units (IU) per mg of meal or protein, were calculated. One trypsin inhibitor unit (TIU) was defined as that which gives a reduction in absorbance at 410 nm of 0.01, relative to trypsin control reactions, in 10 min in a defined assay volume of 10 mL [10]. One chymotrypsin inhibitor unit (CIU) was defined as that which gives a reduction in absorbance at 410 nm of 0.01, relative to chymotrypsin control reactions, in 16 min in a defined assay volume of 10 mL.

### Fractionation of protease inhibitors by cation-exchange chromatography

Finely ground meal (150 mg) was added to 3 mL of 50 mM HCl and stirred for 2 h at 4°C. The extracts were centrifuged at 15,000 *g* for 15 min and supernatants were dialysed extensively against 25 mM sodium acetate buffer, pH 4.4, at 4°C. The protein extracts were fractionated on a MonoS 5/50 GL cation-exchange column. The elution was monitored at 280 nm and 0.5 mL fractions were collected. TIA measurements of eluted proteins were carried out in flat-bottom microtitre plates and assay products measured at OD_405 nm_, as previously described [47]. CIA evaluation of fractionated proteins was carried out, using *N*-benzoyl-L-tyrosine ethyl ester (BTEE), as previously described [46].

### Fractionation of protease inhibitors by size-exclusion chromatography

The oligomerization pattern of pea TI was investigated using albumin proteins extracted from cv. Cameor, wild-type and E109K mutant seeds. Freeze-dried albumins (60 mg dissolved in 3.5 mL 50 mM Tris-HCl pH 7.5) were filtered through a 0.22 μm filter and 2mL samples loaded onto a HiPrep 26/60 Sephacryl S-100 HR column (flow rate of 0.3 ml per min) in 50 mM Tris-HCl, pH7.5. Four replicates samples of each line were analysed. Size calibration of the column separation was based on three sets of standards, each in 2mL buffer [set 1: blue dextran, ovalbumin (48.1 kDa), ribonuclease A (15.6 kDa); set 2: blue dextran, bovine serum albumin (63.5 kDa), chymotrypsinogen A (20.4 kDa); set 3: bovine serum albumin, aprotinin (6.5 kDa)]. Fractions containing TIA were identified, corresponding to three peaks (A, B and C in order of elution from size-exclusion chromatography); these were individually collected, dialysed against distilled water, freeze-dried and dissolved in 3 mL of 50 mM sodium acetate pH 4.4 for analysis by cation-exchange chromatography as above.

### Identification of peptides by mass spectrometric fingerprinting

Following cation-exchange chromatography of some lines, samples from chromatographic peaks having TIA were precipitated with acetone, dissolved in NuPAGE lithium dodecyl sulphate sample buffer (Invitrogen), and the proteins separated by electrophoresis on Novex 12% Bis-Tris pre-cast gels as above. Bands were excised from Colloidal Blue (Invitrogen)-stained gels and subjected to in-gel trypsin digestion. Peptide fragments from digested proteins were desalted and concentrated using C-18 ZipTip columns (Millipore, Madrid, Spain) and then loaded directly onto MALDI plates, using α-cyano-4-hydroxycinnamic acid as the matrix for MALDI mass spectrometry (MS). MS spectra were obtained automatically in a 4700 Proteomics Analyzer (Applied Biosystems, Foster City, CA, USA), operating in reflectron mode with delayed extraction. Peptide mass data were used for protein identification against the MS protein sequence database (www.matrixscience.com).

### Homology modelling

The structural model of pea TI1 was generated using the Phyre2 server (http://www.sbg.bio.ic.ac.uk/phyre2) [50], based on the deposited structure of the pea protein (PDB entry 1PBI) [28] from which it differs by only five amino acid substitutions. The template structure contains a biological homodimer in the crystallographic asymmetric unit and therefore could be used to generate a model of the TI1 homodimer by superposition of two copies of the Phyre2 model. The resultant monomer and dimer models of pea TI1 were not energy-minimised. The interactions at the homodimer interface of TI1 shown in the inset to Fig 6 are identical to those in the template structure. Interactions of Tl1 with its target protein were predicted by superposing the monomer model onto the structure of *Medicago scutellata* BBI (with which it shares 48% amino acid sequence identity) bound to two copies bovine trypsin taken from PDB entry 2ILN [51]. The main part of Fig 6 shows this predicted complex with the structure of *M*. *scutellata* BBI removed.

## Supporting Information

S1 Fig (**a**) Analysis of *TI1* mutants and their corresponding wild-type segregant lines (BC2F3) within each of three mutant families (671, mutation S85F; 895, mutation E109K; 2808, mutation C77Y). Total proteins were extracted from seed meal samples in gel loading buffer and analysed on 12% Bis-Tris gels. M, mutant; WT, wild-type control. The positions of markers are indicated on the left-hand gel (molecular weight x 10–3). (**b**) Total protein content of seeds of *TI1* mutant and wild-type controls (BC2F4), for three mutant families (671, mutation S85F; 895, mutation E109K; 2808, mutation C77Y), using Bradford’s assay. There was no significant difference among lines or mutants (p = 0.05–0.42). (**c**) Analysis of albumin preparations from *TI1* mutants and their corresponding wild-type segregant lines (BC2F3) within each of three mutant families (671, mutation S85F; 895, mutation E109K; 2808, mutation C77Y) on 12% Bis-Tris gels. M, mutant; WT, wild-type control. The positions of markers are indicated on the left-hand gel (molecular weight x 10^−3^). (**d**) Albumin content of seeds of *TI1* mutant and wild-type control lines (BC2F4), for three mutant families (671, mutation S85F; 895, mutation E109K; 2808, mutation C77Y), using Bradford’s assay. There was no significant difference among lines or mutants (p = 0.20–0.36).
(TIF)

S2 Fig Expression of *TI* genes in immature seeds of cv. Cameor, using quantitative PCR of cotyledonary RNA at different stages of development. (**a**) Expression of *TI1* and *TI2* or both, relative to the control gene, *EF1α*, at six stages of increasing maturity (C1–C8). (**b**) Expression of *TI2*, relative to *TI1* at five stages of increasing maturity (C5–C10), where C9 and C10 correspond to stages of maximum protein accumulation. (**c**) Amplification of genomic DNA from two pea genotypes (C, Cameor, J, JI 1294), using two primers designed on *TI1* and *TI2* genes (sense orientation) and I-proof polymerase, alongside DNA markers (M) of up to ~40 kb. Schematic shows *TI1*-*TI2* intergenic region, using gene-specific primers At^YSN^ RC (*TI1*) and At^LSY^ RC (*TI2*) to distinguish gene orientation.
(TIF)

S3 Fig Calibration of the HiPrep 26/60 Sephacryl S-100 column, using five protein standards (bovine serum albumin, ovalbumin, chymotrypsinogen A, ribonuclease A, aprotinin), showing log molecular weight plotted against Kav, determined from elution volume. (TIF)
