# Correction: Eliminating Anti-Nutritional Plant Food Proteins: The Case of Seed Protease Inhibitors in Pea

**DOI:** 10.1371/journal.pone.0138039

**Published:** 2015-09-04

**Authors:** Alfonso Clemente, Maria C. Arques, Marion Dalmais, Christine Le Signor, Catherine Chinoy, Raquel Olias, Tracey Rayner, Peter G. Isaac, David M. Lawson, Abdelhafid Bendahmane, Claire Domoney

There are formatting errors in Table 2. In order to preserve the authors’ original formatting, we have converted the table into a figure. Please see the corrected Table 2 as Fig 1 here.

**Fig 1:**
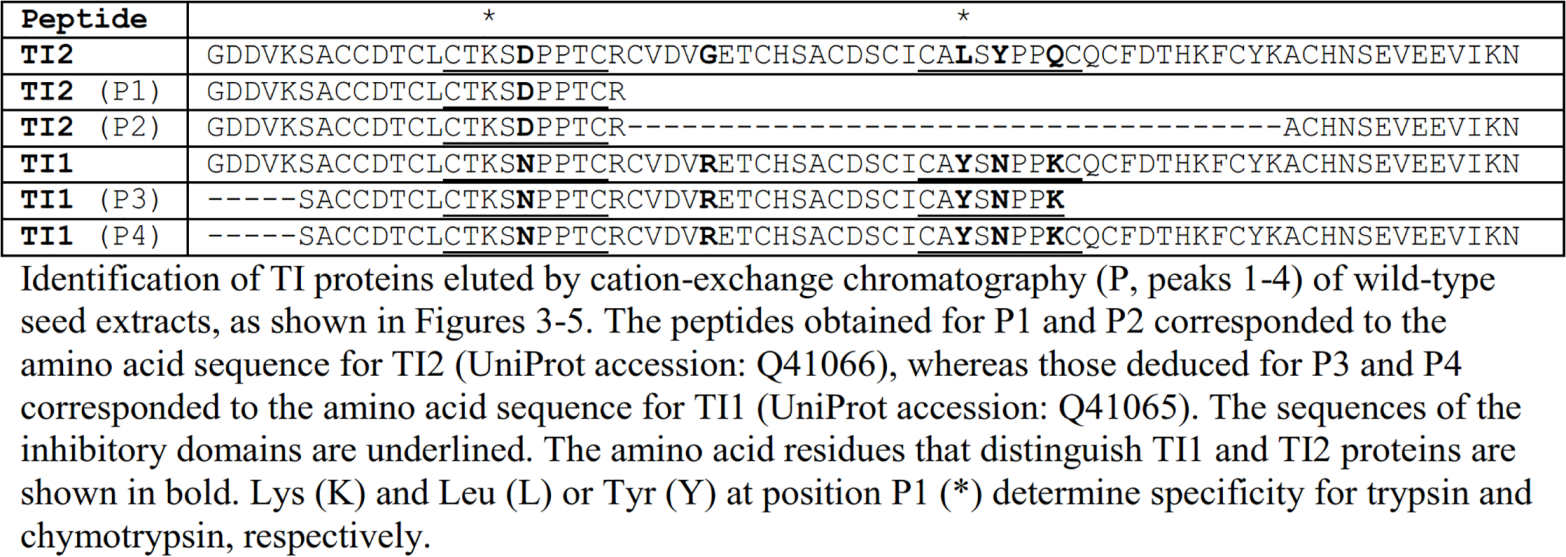
Identification of TI1 and TI2 diagnostic peptides by mass spectrometry.

## References

[1] Clemente A, Arques MC, Dalmais M, Le Signor C, Chinoy C, Olias R, et al (2015) Eliminating Anti-Nutritional Plant Food Proteins: The Case of Seed Protease Inhibitors in Pea. PLoS ONE 10(8): e0134634 doi:10.1371/journal.pone.0134634 26267859 10.1371/journal.pone.0134634 PMC4534040

