# Supplementary figures and images for: Eliminating Anti-Nutritional Plant Food Proteins: The Case of Seed Protease Inhibitors in Pea
Source: PLoS One. 2015 Aug 12;10(8):e0134634. doi: 10.1371/journal.pone.0134634 (PMC4534040; doi:10.1371/journal.pone.0134634)

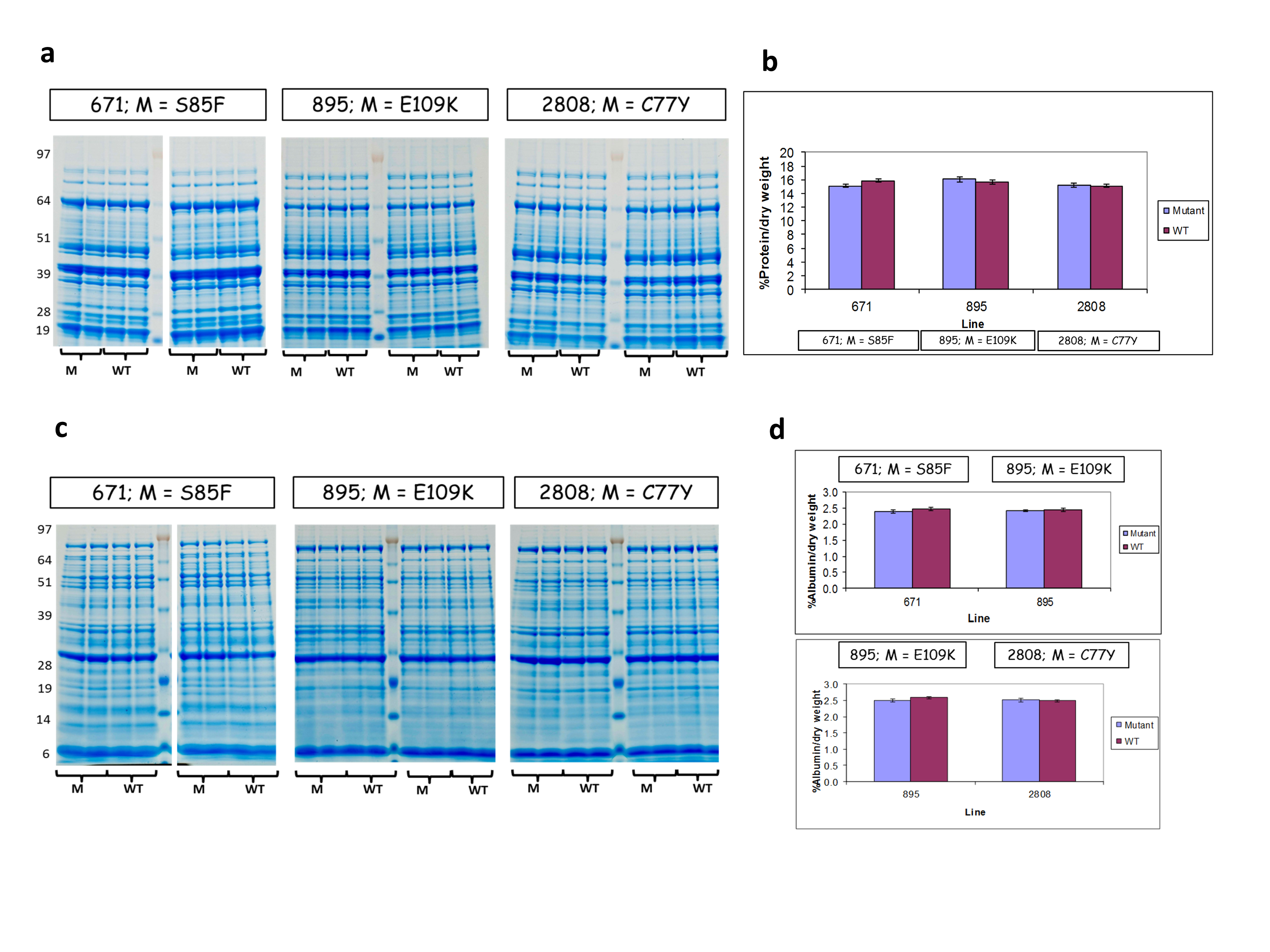

Supplement: S1 Fig — (a) Analysis of TI1 mutants and their corresponding wild-type segregant lines (BC2F3) within each of three mutant families (671, mutation S85F; 895, mutation E109K; 2808, mutation C77Y). Total proteins were extracted from seed meal samples in gel loading buffer and analysed on 12% Bis-Tris gels. M, mutant; WT, wild-type control. The positions of markers are indicated on the left-hand gel (molecular weight x 10–3). (b) Total protein content of seeds of TI1 mutant and wild-type controls (BC2F4), for three mutant families (671, mutation S85F; 895, mutation E109K; 2808, mutation C77Y), using Bradford’s assay. There was no significant difference among lines or mutants (p = 0.05–0.42). (c) Analysis of albumin preparations from TI1 mutants and their corresponding wild-type segregant lines (BC2F3) within each of three mutant families (671, mutation S85F; 895, mutation E109K; 2808, mutation C77Y) on 12% Bis-Tris gels. M, mutant; WT, wild-type control. The positions of markers are indicated on the left-hand gel (molecular weight x 10−3). (d) Albumin content of seeds of TI1 mutant and wild-type control lines (BC2F4), for three mutant families (671, mutation S85F; 895, mutation E109K; 2808, mutation C77Y), using Bradford’s assay. There was no significant difference among lines or mutants (p = 0.20–0.36). (TIF) [file pone.0134634.s001.tif]

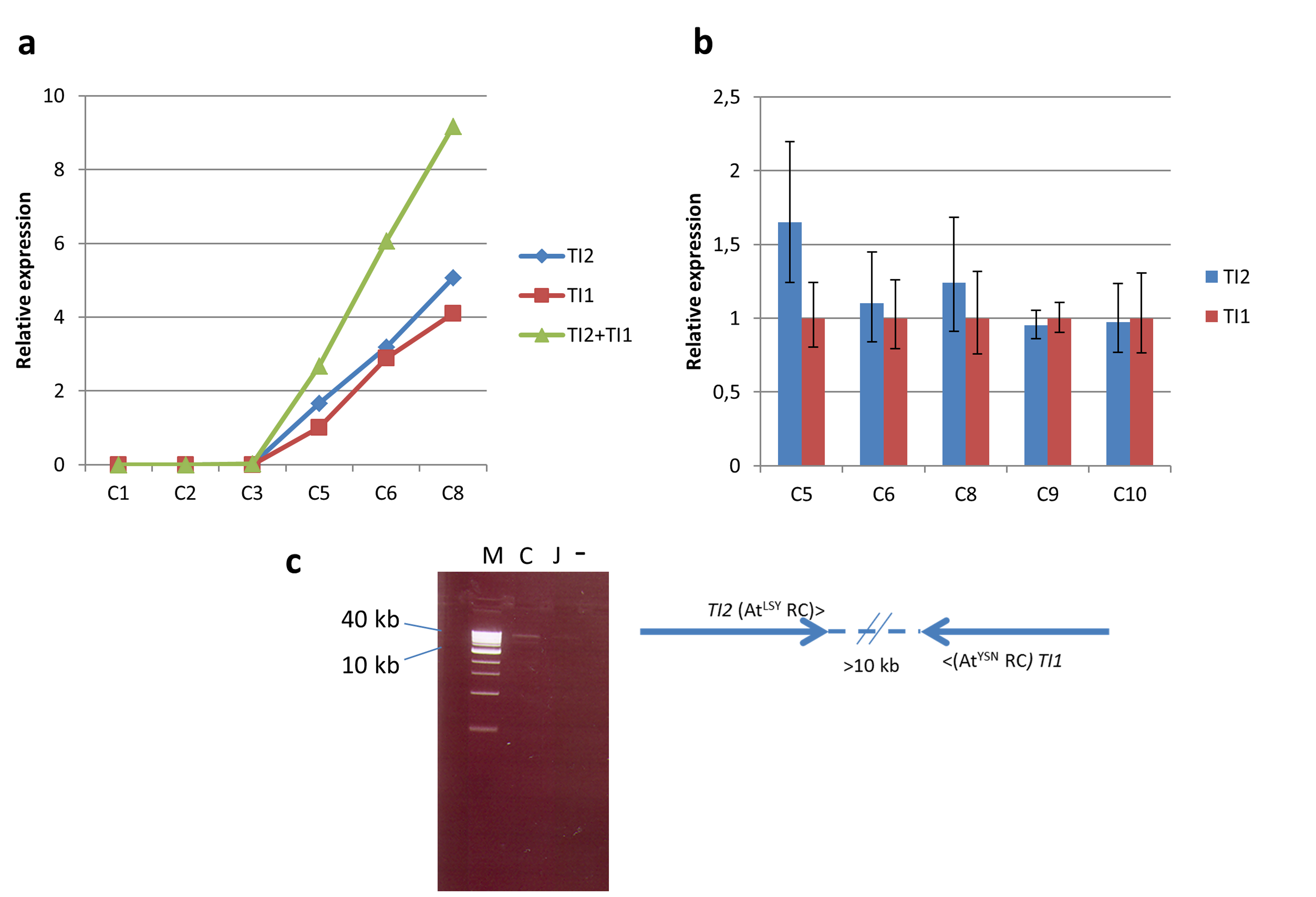

Supplement: S2 Fig — (a) Expression of TI1 and TI2 or both, relative to the control gene, EF1α, at six stages of increasing maturity (C1–C8). (b) Expression of TI2, relative to TI1 at five stages of increasing maturity (C5–C10), where C9 and C10 correspond to stages of maximum protein accumulation. (c) Amplification of genomic DNA from two pea genotypes (C, Cameor, J, JI 1294), using two primers designed on TI1 and TI2 genes (sense orientation) and I-proof polymerase, alongside DNA markers (M) of up to ~40 kb. Schematic shows TI1-TI2 intergenic region, using gene-specific primers AtYSN RC (TI1) and AtLSY RC (TI2) to distinguish gene orientation. (TIF) [file pone.0134634.s002.tif]
